# An evaluation of bone depth at different three-dimensional paths in infrazygomatic crest region for miniscrew insertion: A cone beam computed tomography study

**DOI:** 10.1016/j.heliyon.2024.e25827

**Published:** 2024-02-03

**Authors:** Yingdan Pan, Lijun Wei, Zhanglong Zheng, Wei Bi

**Affiliations:** a Department of Stomatology, Zhongshan Hospital (Xiamen), Fudan University, Xiamen, China

**Keywords:** Orthodontics, Miniscrew, Infrazygomatic crest, Three-dimensional stimulated path, Cone beam computed tomography

## Abstract

**Objective:**

To investigate the difference and distribution of bone depth at different three-dimensional simulated paths to help optimize the insertion path for miniscrew placement in the infrazygomatic crest.

**Methods:**

Cone beam computed tomography scans of 80 adults (38 males and 42 females; mean age, 27.0 years) were assessed. For each subject, bone depth of 81 simulated insertion paths at different insertion points and three-dimensional angulations was measured in 160 infrazygomatic crests; the differences were evaluated using the adjusted Friedman test. The bone deficiency ratio for each path was calculated. Distributions of measurements were analyzed and reported as specially designed colormaps.

**Results:**

Bone depth increased, and bone deficiency ratio reduced mesially to distally (P < 0.001), apically to coronally (P < 0.01), and at a greater gingival and distal inclination (P < 0.05). The maximum bone depth (10.72 mm) was observed 13 mm above the maxillary occlusal plane in the mesiobuccal root of the maxillary second molar. The minimum bone depth (3.4 mm) was observed 17 mm above the maxillary occlusal plane in the distobuccal root of the maxillary first molar. No bone deficiency was detected at the paths of 13 mm above the maxillary occlusal plane at a gingival inclination of 70° and distal inclination of 30° in the mesiobuccal root of the maxillary second molar. The highest bone deficiency ratio is present 17 mm above the maxillary occlusal plane at a gingival inclination of 60° and a distal inclination of 0° in the distobuccal root of the maxillary first molar (89/160).

**Conclusion:**

Insertion paths located at 13 mm above the maxillary occlusal plane in the mesiobuccal root of the maxillary second molar were optimal. A gingival inclination of 70° and a distal inclination of 30° could be beneficial. The distobuccal root of the maxillary first molar region or above the 17 mm insertion plane may not be recommended.

## Highlights

• Measuring bone depth at different three-dimensional simulated paths.
• Distributions of bone measurements were reported as specially designed colormaps.
• Mesiobuccal root of the maxillary second molar is suitable for miniscrew insertion.
• A distal inclination of 30° for miniscrew in infrazygomatic crest is beneficial.

## Graphical abstract

**Figure:**
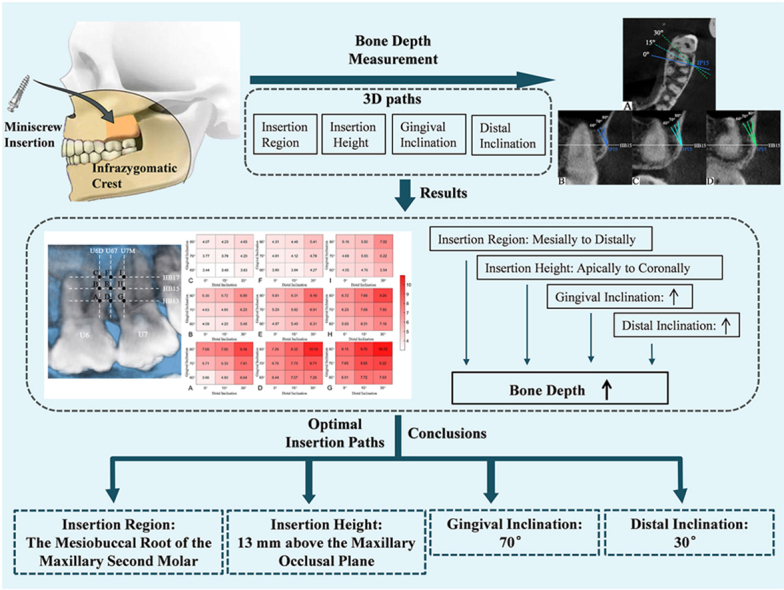

## 1 Background

Miniscrew implants are widely applied as orthodontic temporary skeletal anchorage devices, which can provide absolute anchorage and greatly improve orthodontic treatment [1,2]. The infrazygomatic crest (IZC) region has become a commonly used extra-alveolar site for miniscrew insertion in the maxilla because of the thickest maxillary cortical bone and its position beyond the dentoalveolar area, which provides better prior stability and unobstructed tooth movement [3,4]. Thus, the IZC miniscrew is frequently applied for total arch maxillary distalization in the treatment of Class II malocclusion to achieve good therapeutic effects [5,6]. Nonetheless, the difficulty and risks of miniscrew insertion operation in the IZC region cannot be ignored.

Intraorally, the IZC is located lateral to the roots of the maxillary first molar (U6) and the maxillary second molar (U7) [7,8]. Due to the thin buccal alveolar bone and the deeper sinus floor, maxillary sinus perforation and buccal root injury may occur during insertion, increasing the risk of infection in the maxillary sinus and loosening of the miniscrews [9,10]. This, coupled with the insertion site located in the back of the mouth, brings challenges to many orthodontists. Buccal alveolar bone availability is a critical factor for the placement of IZC miniscrews, which determines the insertion method and influences success rates [11]. Consequently, it is important to understand the anatomical dimensions of this region and design the three-dimensional (3D) insertion path of miniscrew thoroughly to increase bone contact and avoid damage to the surrounding anatomical structures.

Multiple studies have used cone beam computed tomography (CBCT) analysis to assess the buccal bone in the IZC [[12], [13], [14], [15], [16], [17], [18], [19]]. However, most of these have only measured buccal bone thickness or bone depth in a constant two-dimensional section and simply addressed the impact of insertion height and gingival inclination on bone depth. In common insertion procedures in the IZC region, the insertion path is prone to involuntarily incline distally due to the restricted placement of the operating handle by the limited width of the mouth. Thus, the impact of this distal inclination of the insertion path should be included in analyses [20]. Recently, Du et al. [20] used 3D reconstruction models to explore bone depths and thicknesses of different insertion paths with different gingival and distal tipping angles in the IZC. However, the insertion points of their study were only set at the midpoints between the roots of U6 and U7. Temple et al. [21] found that the buccal bone plate increased distally with maximum values in the mesiobuccal root of the maxillary second molar (U7M). The appropriate insertion positions and angles, including gingival and distal inclination, for the IZC miniscrew are still controversial and have not been well documented in the U7M region.

The accuracy of CBCT for the measurement of alveolar bone has been confirmed [22]. This study aimed to use CBCT to simulate the 3D insertion paths at different gingival and distal inclinations from multiple insertion points in the IZC region, comprehensively evaluate the bone depth of different paths from the distobuccal root of the maxillary first molar (U6D) to U7M and explore the differences and distributions of bone depth and bone deficiency ratio. Our objective was to locate the optimal insertion paths for IZC miniscrew placement and identify multiple alternative insertion options for cases in which reoperation for fixation of miniscrew may be needed.

## 2 Methods and materials

### 2.1 Study sample

This retrospective study was approved by the appropriate Institutional Review Board (No. B2022-012), and followed the principles in the Declaration of Helsinki. All CBCT scans were taken from May 2020 to January 2022, purely for clinical examination and treatment planning before impacted tooth extraction, orthodontic treatment, or other oral treatment. Written informed consent was signed by each participant, allowing their CBCT images to be used for research purposes.

CBCT data were selected for this study from a total of 80 adult patients (38 males and 42 females; mean age, 27 years; range, 19–38 years) who met the following inclusion criteria: (1) no missing or supernumerary tooth in the maxillary arch; (2) no crowding of U6 and U7; (3) no radiographic signs of periodontal disease; (4) no craniofacial abnormalities or history of trauma; (5) no orthodontic treatment history; and (6) clear CBCT images.

### 2.2 Image acquisition

All CBCT scans were obtained by an experienced technician using a CBCT apparatus (Sirona Orthophos SL 3D, Bensheim, Germany) at an 11*10-cm field of view, 85 kV, 10 mAs, and a scan time of 5000 ms; the resulting voxel was 0.16 mm. Routine calibration and system quality assurance tests of the CBCT device have been performed regularly. The patients examined were positioned in the Frankfort plane parallel to the floor using a cephalostat. CBCT data were exported to the standard dicom format, and 3D images were reconstructed and evaluated using SIDEXIS 4 Imaging Software (Anatomage, San Jose, California, USA) with the personal information anonymized and de-identified.

### 2.3 Pre-analysis image adjustment

**Fig. 1:**
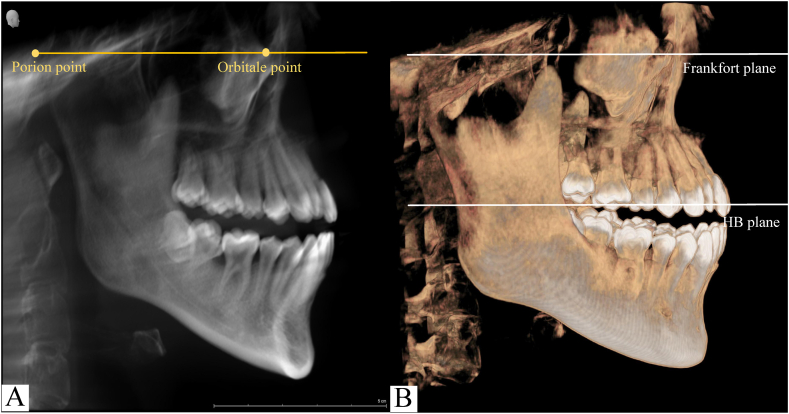
Construction of the horizontal base (HB) plane. (A) Standardized head position with the Frankfort horizontal plane parallel to the bottom edge of the screen in the cephalometrics interface. (B) The HB plane established parallel to the Frankfort horizontal plane and aligned with the mesiobuccal cusps of both maxillary first molars.

Images were first adjusted in brightness, contrast, and zoom for better visualization of hard tissue. Furthermore, they were preliminarily oriented to ensure that the Frankfort horizontal plane was parallel to the bottom edge of the screen using the orbital and porion points as landmarks in the cephalometrics interface to obtain a standardized head position (Fig. 1A). Simultaneously, the FMA (Frankfurt mandibular plane angle) of each participant was measured in the cephalometics interface to ensure that the skeletal growth pattern of the included subjects is normodivergent (21° <FMA <29°) [19]. Subsequently, by moving the axial section line, the horizontal base (HB) plane was established as the maxillary occlusal plane, a plane aligned with the U6 mesiobuccal cusps and parallel to the Frankfort horizontal plane (Fig. 1B) [14].

**Fig. 2:**
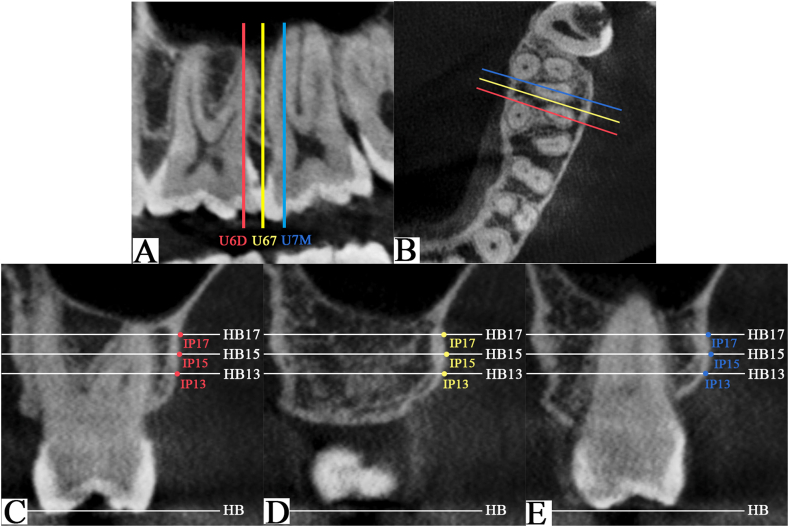
Construction of the measured sections at the left side. (A) Sagittal section: location of the three studied coronal slices [distobuccal root of the maxillary first molar (U6D), between the maxillary first molar and the maxillary second molar (U67), and mesiobuccal root of the maxillary second molar (U7M)]. (B) Axial section: location of the three studied coronal slices. Definition of the three insertion heights: 13 mm, 15 mm, and 17 mm above the horizontal base (HB) plane (HB13, HB15, HB17) and insertion points (IP) at 13 mm (IP13), 15 mm (IP15), and 17 mm (IP17) above the horizontal base plane in U6M (C), U67(D) and U7M(E) sections, respectively.

The coronal, sagittal, and axial sections were used to display and orient the image of the IZC regions to accurately locate the three coronal slices of interest, which are perpendicular to the surface of the buccal bone (Fig. 2A and B): one slice in the long axis of U6D, one in the long axis of U7M, and another slice passing through the interproximal contact between the crowns of U6 and U7 (U67). Three vertical heights—13, 15, and 17 mm—above the HB were established in these three coronal slices, respectively (HB13, HB15, and HB17) (Fig. 2C–E). This procedure was accomplished by moving the horizontal reference line according to the millimeter rule at the frame border.

### 2.4 Generation of simulated paths and measurement of bone depth

**Fig. 3:**
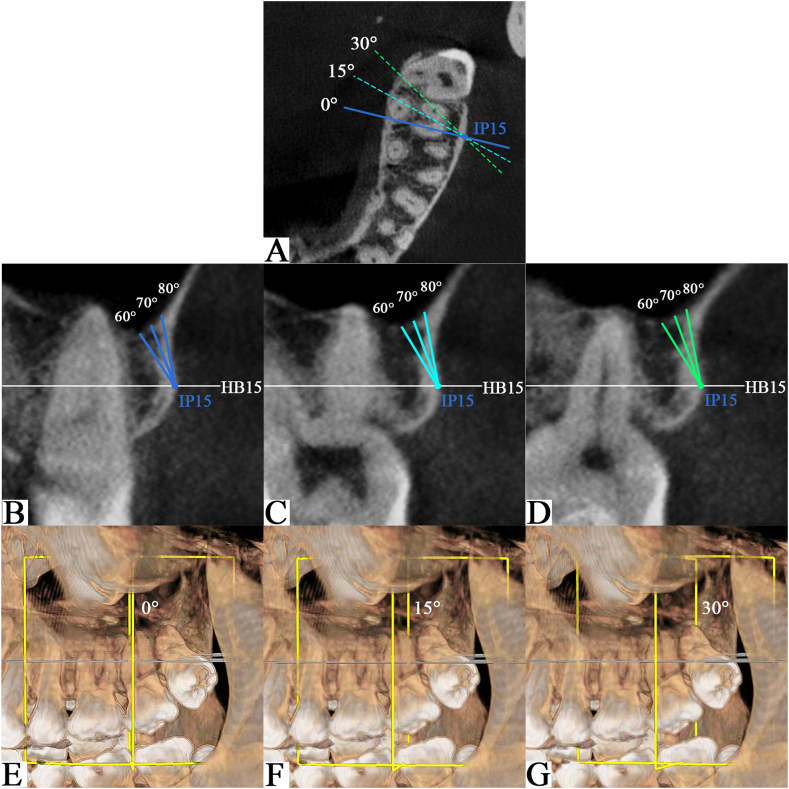
Measurements of bone depth at 15 mm above the horizontal base (HB) plane (HB15) in the mesiobuccal root of the maxillary second molar (U7M). (A) Construction of three measured coronal slices in the axial section through the insertion point (IP) at 15 mm above the horizontal base plane (IP15). Dotted lines illustrating the three measured coronal slices with additional distally rotated angles of 0°, 15°, and 30°. Measurements of bone depth at gingival inclination of 60°, 70°, and 80° with 0°(B), 15°(C), and 30°(D) distal inclinations in three generated slices. Three-dimensional reconstruction interfaces showing the generation of three measured coronal slices at 0°(E), 15°(F), and 30°(G) of distal rotation.

In any of the three coronal slices mentioned above, the intersections between the three vertical lines (HB13, HB15, and HB17) and the surface of the buccal bone defined the miniscrew insertion points (IP13, IP15, and IP17) (Fig. 2C–E). For each IP, three lines were drawn passing through the IP in gingival direction at three different angles (60°, 70°, and 80°). The three bone depth measurements were made along these lines, from the IP to the points where the lines touch the sinus wall or root (Fig. 3B).

Then, we used the function of rotatable sections in CBCT to rotate the studied coronal slices at 15° and 30° in the distal direction on the axis of the vertical line crossing the IP (Fig. 3A); thus, a total of three coronal slices for each IP were obtained. The bone depth at the gingival inclination of 60°, 70°, and 80° was then measured in three generated coronal slices(Fig. 3B–D), which meant that additional distal inclinations at 0°, 15° and 30° were added to the insertion path(Fig. 3E–G). Therefore, nine bone depth values could be measured from each IP. The measurement was repeated for three IPs (IP13, IP15, and IP17) in each coronal slice to obtain 27 measurements of bone depth. Finally, a total of 81 measurements could be obtained in the three coronal slices studied (U6D, U67, and U7M) in an IZC region. Measurements were obtained on both left and right sides of the maxilla.

The minimum bone depth of the miniscrew is recommended to be 3.8 mm for prolonged stability [20,23]. Thus, we defined bone deficiency as a bone depth <3.8 mm.

Twenty CBCT images were randomly selected and measured twice at an interval of four weeks by the same operator under identical conditions. The intraclass correlation coefficient (ICC) was calculated to evaluate the intra-examiner agreement between the measurements.

### 2.5 Statistical analysis

All statistical analyses were performed using SPSS 23.0 (IBM, Armonk, NY, USA). The statistical significance for this study was set at 5%. According to the Shapiro–Wilk normality test, the data were not normally distributed with homogeneous variance. The bone depth measurements were expressed as median (25th–75th percentile) and mean ± standard deviation (SD) simultaneously. The Mann–Whitney *U* test was applied to detect the measurement differences between sexes and the Wilcoxon signed rank test was performed to compare the measurements between the two sides. Comparisons of bone depth between different insertion regions, height, and simulated insertion inclinations were made using the Friedman test, and the significance level was adjusted by Bonferroni correction with Tukey's post hoc test for multiple tests. Each pairwise test was performed between only one variable with other variables controlled.

The bone deficiency ratio was calculated for each insertion path. Lastly, special color maps were designed to display the distribution of bone depth and bone deficiency ratio of each simulated path.

## 3 Results

The ICC values ranged from 0.889 to 0.976, indicating good to excellent intra-examiner reproducibility for repeated measures. The bone depth measurements between the sexes and sides showed no statistically significant differences (P > 0.05) (Tables S1 and S2), and all participants presented a normodivergent growth pattern (FMA values ranged from 21.4° to 28.5°, mean FMA±SD: 24.99° ± 1.79°). Therefore, all measurements from the 160 sites were pooled for the following evaluation.

In keeping with the conventional clinical practice of aligning the operating handle at 0° of distal inclination, the bone depths at different insertion heights and gingival inclinations in the three insertion regions, with the distal inclination set at 0° are shown in Table 1. From U6D to U67 to U7M, bone depth increased significantly (P < 0.001) at the same insertion height and gingival inclination, except between U6 and U67 at HB17 at a gingival inclination of 70° and 80° (P > 0.05).

**Table 1:** Bone depth (mm) at different insertion heights and different gingival inclinations with 0° of distal inclination in three insertion regions (n = 160).

Insertion height	Gingival inclination	Insertion region					
		U6D		U67		U7M	
		Median (25th-75th)	Mean ± SD	Median (25th-75th)	Mean ± SD	Median (25th-75th)	Mean ± SD
HB13	60°	3.96 (3.15–5.20)	4.24 ± 1.79	6.44 (5.25–8.19)^a^	7.00 ± 2.87	6.51 (5.34–7.75)^ab^	6.71 ± 2.09
	70°	5.71 (4.26–7.31)^e^	5.90 ± 2.48	6.78 (5.58–8.49)^a^	7.33 ± 2.63	7.65 (6.24–9.37)^ab,e^	7.94 ± 2.49
	80°	7.08 (5.42–8.58)^ef^	7.26 ± 2.87	7.29 (6.05–9.06)^a^,^ef^	7.86 ± 2.66	8.15 (6.80–10.15)^ab, ef^	8.58 ± 2.65
HB15	60°	4.09 (3.11–5.25)	4.48 ± 2.25	4.97 (4.10–6.73)^a^,^c^	5.77 ± 2.81	5.63 (4.55–7.16)^ab^,^c^	6.24 ± 2.60
	70°	4.63 (3.65–6.16)^c^,^e^	5.19 ± 2.44	5.29 (4.32–7.00)^a^,^c^	5.89 ± 2.52	6.23 (4.89–8.21)^ab^,^c^	6.66 ± 2.50
	80°	5.38 (4.31–7.01)^c, ef^	5.88 ± 2.73	5.81 (4.60–7.48)^a^,^c^,^e^	6.24 ± 2.61	6.72 (5.21–8.68)^ab,c, ef^	7.08 ± 2.62
HB17	60°	3.44 (2.74–4.68)^cd^	4.17 ± 2.46	3.90 (2.91–5.11)^a, cd^	4.41 ± 2.41	4.55 (3.36–6.17)^ab, cd^	4.92 ± 2.29
	70°	3.77 (2.80–5.13)^cd^	4.34 ± 2.36	4.01 (3.03–5.39)^cd^	4.50 ± 2.25	4.68 (3.53–6.42)^ab, cd^	5.10 ± 2.24
	80°	4.07 (2.96–5.49)^cd, ef^	4.64 ± 2.48	4.31 (3.23–5.68)^cd, ef^	4.78 ± 2.35	5.61(3.74–6.78)^ab, cd, ef^	5.45 ± 2.40

U6D, distobuccal root of the maxillary first molar; U67, between the maxillary first molar and the maxillary second molar (U67); U7M, mesiobuccal root of the maxillary second molar; HB, horizontal base; HB13, 13 mm above the horizontal base plane; HB15, 15 mm above the horizontal base plane; HB17, 17 mm above the horizontal base plane; SD, standard deviation.

Note: Bone depth was compared between the variables when the other two variables were controlled.

^a^Significantly different from the U6D group at the same insertion height and gingival inclination.

^b^Significantly different from the U67 group at the same insertion height and gingival inclination.

^c^Significantly different from the HB13 group at the same gingival inclination in the same insertion region.

^d^Significantly different from the HB15 group at the same gingival inclination in the same insertion region.

^e^Significantly different from the 60° group at the same insertion height in the same insertion region.

^f^Significantly different from the 70° group at the same insertion height in the same insertion region.

For any of the three regions, the lower the insertion height and the larger the gingival inclination, the greater the bone depth. At the same gingival inclination, a significant difference was found between any two insertion heights (P < 0.01) except between HB13 and HB15 at a gingival inclination of 60° in U6D (P > 0.05). At the same insertion height, a significant difference was found between any two gingival inclinations (P < 0.05) except between 60° and 70° at HB17 in U6D; at HB13, HB15, and HB17 in U67; and at HB15 and HB17 in U7M and between 70° and 80° at HB15 in U67 (P > 0.05).

The impact of additional distal inclination on bone depth in different paths is shown in Table 2. Bone depth increased with greater distal inclination (P < 0.05), but no significant difference was found between 0° and 15° at HB17 in U6D and U67 and between 15° and 30° at a gingival inclination of 60° at HB13 in U7M (P > 0.05). The maximum bone depth (median, 10.72 mm; mean 11.69 mm) was observed at HB13 at a gingival inclination of 80° and a distal inclination of 30° in U7M. The minimum bone depth (median, 3.4 mm; mean, 4.17 mm) was observed at HB17 at a gingival inclination of 60° and a distal inclination of 15° in U6D.

**Table 2:** Comparison of bone depth (mm) among insertion paths at distal inclinations of 0°, 15° and 30° (n = 160).

Insertion region	Insertion height	Gingival inclination	Distal inclination					
			0°		15°		30°	
			Median (25th-75th)	Mean ± SD	Median (25th-75th)	Mean ± SD	Median (25th-75th)	Mean ± SD
U6D	HB13	60°	3.96 (3.15–5.20)	4.24 ± 1.79	4.93 (3.65–6.31)^a^	5.27 ± 2.47	6.64 (4.45–9.04)^ab^	7.07 ± 3.56
		70°	5.71 (4.26–7.31)	5.90 ± 2.48	6.33 (4.76–8.48)^a^	6.78 ± 3.04	7.91 (6.30–10.61)^ab^	8.49 ± 3.62
		80°	7.08 (5.42–8.58)	7.26 ± 2.87	7.55 (6.08–9.49)^a^	7.97 ± 3.16	9.16 (7.11–12.08)^a^^,^^b^	9.64 ± 3.80
	HB15	60°	4.09 (3.11–5.25)	4.48 ± 2.25	4.20 (3.25–5.74)^a^	4.90 ± 2.67	5.48 (3.66–7.27)^a^^,^^b^	5.99 ± 3.31
		70°	4.63 (3.65–6.16)	5.19 ± 2.44	4.85 (3.65–6.75)^a^	5.58 ± 2.85	6.23 (4.16–7.97)^a^^,^^b^	6.57 ± 3.36
		80°	5.38 (4.31–7.01)	5.88 ± 2.73	5.72 (4.18–7.49)^a^	6.16 ± 2.92	6.89 (5.03–9.17)^a,^^b^	7.41 ± 3.57
	HB17	60°	3.44 (2.74–4.68)	4.17 ± 2.46	3.40 (2.62–4.83)	4.17 ± 2.59	3.83 (2.64–5.58)^ab^	4.59 ± 2.87
		70°	3.77 (2.80–5.13)	4.34 ± 2.36	3.78 (2.76–5.09)	4.36 ± 2.54	4.20 (2.91–6.07)^ab^	4.98 ± 3.11
		80°	4.07 (2.96–5.49)	4.64 ± 2.48	4.23 (2.91–5.64)	4.69 ± 2.67	4.65 (3.20–6.69)^ab^	5.43 ± 3.28
U67	HB13	60°	6.44 (5.25–8.19)	7.00 ± 2.87	7.07 (5.50–9.13)^a^	7.63 ± 3.12	7.25 (5.99–8.75)^ab^	7.67 ± 2.69
		70°	6.78 (5.58–8.49)	7.33 ± 2.63	7.73 (6.08–9.55)^a^	8.20 ± 2.79	8.71 (7.23–10.74)^ab^	9.50 ± 3.56
		80°	7.29 (6.05–9.06)	8.20 ± 2.79	8.32 (6.60–10.70)^a^	8.89 ± 3.11	10.15 (8.28–12.80)^ab^	10.80 ± 3.89
	HB15	60°	4.97 (4.10–6.73)	4.48 ± 2.25	5.49 (4.28–7.43)^a^	6.14 ± 2.98	6.31 (4.63–8.12)^ab^	6.80 ± 3.09
		70°	5.29 (4.32–7.00)	5.19 ± 2.44	5.82 (4.53–7.81)^a^	6.34 ± 2.78	6.91 (5.09–9.49)^ab^	7.62 ± 3.59
		80°	5.81 (4.60–7.48)	5.88 ± 2.73	6.31 (4.77–8.54)^a^	6.84 ± 2.96	8.10 (5.63–10.60)^a^^,^^b^	8.45 ± 3.83
	HB17	60°	3.90 (2.91–5.11)	4.17 ± 2.46	3.84 (2.85–5.46)	4.51 ± 2.64	4.27 (3.01–6.54)^a^^,^^b^	5.09 ± 3.03
		70°	4.01 (3.03–5.39)	4.34 ± 2.36	4.12 (2.95–5.78)	4.69 ± 2.51	4.78 (3.24–7.18)^a^^,^^b^	5.51 ± 3.32
		80°	4.31 (3.23–5.68)	4.64 ± 2.48	4.48 (3.22–6.59)	5.06 ± 2.68	5.41 (3.57–8.08)^a^^,^^b^	6.03 ± 3.38
U7M	HB13	60°	6.51 (5.34–7.75)	6.71 ± 2.09	7.72 (5.77–8.95)^a^	7.77 ± 2.71	7.63 (5.85–9.39)^a^	8.08 ± 3.51
		70°	7.65 (6.24–9.37)	7.94 ± 2.49	8.93 (7.46–11.06)^a^	9.14 ± 2.76	9.22 (7.66–11.56)^a^^,^^b^	10.18 ± 4.13
		80°	8.15 (6.80–10.15)	8.58 ± 2.65	9.70 (7.69–12.19)^a^	10.01 ± 3.20	10.72 (8.89–14.05)^a^^,^^b^	11.69 ± 3.95
	HB15	60°	5.63 (4.55–7.16)	6.24 ± 2.60	6.31 (4.84–8.26)^a^	6.80 ± 3.76	7.16 (5.50–8.72)^a,b^	7.81 ± 3.62
		70°	6.23 (4.89–8.21)	6.66 ± 2.50	7.08 (5.40–9.12)^a^	7.37 ± 2.83	7.93 (6.42–10.37)^a^^,^^b^	8.84 ± 3.79
		80°	6.72 (5.21–8.68)	7.08 ± 2.62	7.68 (5.90–9.91)^a^	8.04 ± 3.08	9.25 (7.01–11.79)^a^^,^^b^	9.84 ± 3.94
	HB17	60°	4.55 (3.36–6.17)	4.92 ± 2.29	4.76 (3.30–6.64)	5.20 ± 2.50	5.54 (3.75–7.79)^a^^,^^b^	6.32 ± 3.56
		70°	4.68 (3.53–6.42)	5.10 ± 2.24	5.03 (3.61–7.05)^a^	5.52 ± 2.67	6.22 (4.03–8.75)^ab^	6.90 ± 3.78
		80°	5.16(3.74–6.78)	5.45 ± 2.40	5.50 (3.94–7.81)^a^	6.04 ± 2.87	7.02 (4.47–9.80)^ab^	7.58 ± 3.84

U6D, distobuccal root of the maxillary first molar; U67, between the maxillary first molar and the maxillary second molar (U67); U7M, mesiobuccal root of the maxillary second molar; HB, horizontal base; HB13, 13 mm above the horizontal base plane; HB15, 15 mm above the horizontal base plane; HB17, 17 mm above the horizontal base plane; SD, standard deviation.

a Significantly different from the 0° group at the same insertion height and gingival inclination in the same insertion region.

b Significantly different from the 15° group at the same insertion height and gingival inclination in the same insertion region.

**Fig. 4:**
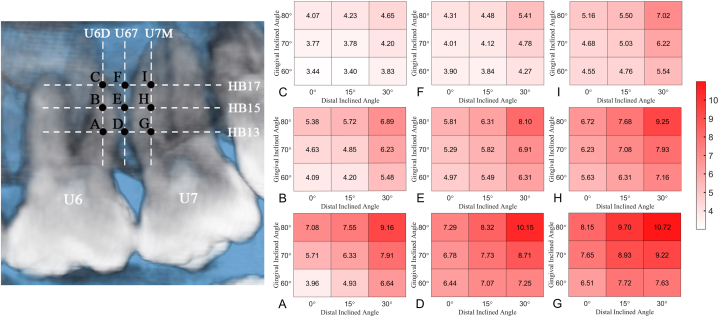
Median of bilateral bone depth (mm) map of each insertion path at 13 mm, 15 mm, and 17 mm above the horizontal base (HB) plane (HB13, HB15, HB17) in distobuccal root of the maxillary first molar (U6D), between the maxillary first molar and the maxillary second molar (U67) and mesiobuccal root of the maxillary second molar (U7M), respectively. Capital letters (A to I) represent different insertion points. Every single color-map indicates the bone depth distribution in the assigned insertion point at different gingival and distal inclinations. (For interpretation of the references to color in this figure legend, the reader is referred to the Web version of this article.)

Fig. 4(A-I) shows the color maps of the median values of bone depth in all paths. Bone depth increased mesially to distally, apically to coronally, and at a greater gingival and distal inclination.

**Fig. 5:**
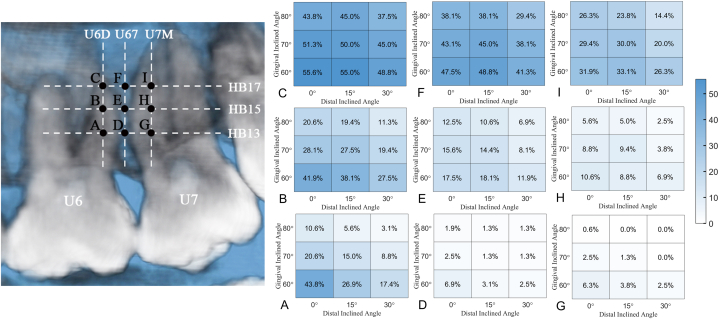
Bilateral ratio of bone deficiency (%) map of each insertion path at 13 mm, 15 mm, and 17 mm above the horizontal base (HB) plane (HB13, HB15, HB17) in distobuccal root of the maxillary first molar (U6D), between the maxillary first molar and the maxillary second molar (U67) and mesiobuccal root of the maxillary second molar (U7M), respectively. Capital letters (A to I) represent different insertion points. Every single color-map indicates the bone deficiency ratio distribution in the assigned insertion point at different gingival and distal inclinations. (For interpretation of the references to color in this figure legend, the reader is referred to the Web version of this article.)

The bone deficiency ratio for different insertion paths is shown in Table 3 and Fig. 5(A-I). There was a general tendency for the bone deficiency ratio to decline from U6M to U67 to U7M, with a decrease in the insertion height and an increase in the gingival and distal inclinations. No bone deficiency was found at HB13 at a gingival inclination of 70° and a distal inclination of 30° or at a gingival inclination of 80° and a distal inclination of 15° and 30° in U7M. The highest bone deficiency ratio is present at HB17 at a gingival inclination of 60° and a distal inclination of 0° in U6D (89/160).

**Table 3:** Numbers and ratio of bone deficiency of different insertion paths (n = 160).

Insertion height	Gingival inclination	Distal inclination	Insertion region					
			U6D		U67		U7M	
			Number	Ratio(%)	Number	Ratio(%)	Number	Ratio(%)
HB13	60°	0°	70/160	43.8	11/160	6.9	10/160	6.3
		15°	43/160	26.9	5/160	3.1	6/160	3.8
		30°	28/160	17.4	4/160	2.5	4/160	2.5
	70°	0°	33/160	20.6	4/160	2.5	4/160	2.5
		15°	24/160	15.0	2/160	1.3	2/160	1.3
		30°	14/160	8.8	2/160	1.3	0/160	0.0
	80°	0°	17/160	10.6	3/160	1.9	1/160	0.6
		15°	9/160	5.6	2/160	1.3	0/160	0.0
		30°	5/160	3.1	2/160	1.3	0/160	0.0
HB15	60°	0°	67/160	41.9	28/160	17.5	17/160	10.6
		15°	61/160	38.1	29/160	18.1	14/160	8.8
		30°	44/160	27.5	19/160	11.9	11/160	6.9
	70°	0°	45/160	28.1	25/160	15.6	14/160	8.8
		15°	44/160	27.5	23/160	14.4	15/160	9.4
		30°	31/160	19.4	13/160	8.1	6/160	3.8
	80°	0°	33/160	20.6	20/160	12.5	9/160	5.6
		15°	31/160	19.4	17/160	10.6	8/160	5.0
		30°	18/160	11.3	11/160	6.9	4/160	2.5
HB17	60°	0°	89/160	55.6	76/160	47.5	51/160	31.9
		15°	88/160	55.0	78/160	48.8	53/160	33.1
		30°	78/160	48.8	66/160	41.3	42/160	26.3
	70°	0°	82/160	51.3	69/160	43.1	47/160	29.4
		15°	80/160	50.0	72/160	45.0	48/160	30.0
		30°	72/160	45.0	61/160	38.1	32/160	20.0
	80°	0°	70/160	43.8	61/160	38.1	42/160	26.3
		15°	72/160	45.0	61/160	38.1	38/160	23.8
		30°	60/160	37.5	47/160	29.4	23/160	14.4

U6D, distobuccal root of the maxillary first molar; U67, between the maxillary first molar and the maxillary second molar (U67); U7M, mesiobuccal root of the maxillary second molar; HB, horizontal base; HB13, 13 mm above the horizontal base plane; HB15, 15 mm above the horizontal base plane; HB17, 17 mm above the horizontal base plane.

## 4 Discussion

Although studies have demonstrated that maxillary sinus perforation does not compromise the stability of IZC miniscrews [24,25], adequate bone depth must be gained during insertion to limit the sinus penetration depth and prevent root contact in the IZC region to minimize operation risk [14]. This study explored bone depth and the bone deficiency ratio of different postulated insertion paths in three dimensions for selection and optimization of the clinical IZC miniscrew insertion protocol.

Consistent with previous studies [4,12,14,16,20], our results showed no difference between the sexes. Moreover, no significant difference was found between the left and right sides, which might be owing to the facial symmetry and relatively well aligned U6 and U7 in the patients included in this study. To our knowledge, skeletal growth pattern is related to different alveolar bone structures, but the relationship between skeletal growth pattern and bone thickness of the IZC remains controversial [16,19,26]. To ensure homogeneity, all the included participants in this study have a normodivergent growth pattern. Patients with various growth patterns should be examined in further studies to validate the findings.

Distal inclination was set at 0° when the first group of comparisons was performed between different insertion regions (U6D, U67, and U7M), different insertion heights (HB13, HB15, and HB17), and different gingival inclinations (60°, 70°, and 80°), as the outcomes can be mainly attributed to the thickness of the buccal bone and the level of the maxillary sinus floor in the absence of other confounders.

Bone depth tended to increase from U6D to U7M at the same insertion height and inclination. Although few studies have investigated the bone depth in the U7M region, thicker buccal alveolar bone has been reported [21,27], which can probably explain our findings. Previous studies considered U6 as a common region for insertion in the IZC [9,12,14,16], but in recent years, growing research has focused on region U67 instead for rich bone mass [17,18,20]. However, there is a risk that the miniscrew might injure or block the root of U6 if it is inserted into the narrow radicular region of U67, especially during the molar distalization movement. In clinical practice, the evaluation of the distance between U6 and U7 before insertion cannot be ignored. The mean interradicular distance of the U67 buccal aspect increased apically and ranged from 2.65 ± 0.88 to 4.05 ± 1.04 between the level of 5–11 mm above the alveolar bone crest [18]. Proper increase of insertion height to avoid root contact in the narrow U67 region is acceptable. Alternatively, based on our findings, U7M can be considered as an applicable extra-alveolar region with sufficient bone depth for insertion.

Besides the insertion region, the height of the insertion and the gingival slope can directly affect bone depth [9]. We found that as the height of the insertion increased, bone depth tended to decrease and the ratio of bone deficiency increased, which was in agreement with previous studies [12,16,17]. Regarding soft tissue, high degree of insertion at the alveolar mucosa will frequently cause mucosal inflammation, soft tissue embedment, or patient discomfort. Furthermore, the mucogingival junction located approximately 13 mm above the maxillary occlusal plane may have less mucogingival risk [28]; therefore, insertion paths at HB13 in U67 and U7M are recommended since the ratio of bone deficiency of all these paths is <10%. In the U6D region, we found an abnormally shallow bone depth and a high bone deficiency ratio at HB13 and HB15 at a gingival inclination of 60°. It seems because the insertion path was obstructed by distobuccal root of U6 covered by continuous thin bone plate. This condition can be improved when gingival inclination rises, corroborating the findings reported by Liou et al. [14]; however, slippage of the miniscrew and bone stripping may occur during insertion when gingival inclination of 80° is performed [14]. Moreover, taking the diameter of the miniscrew into consideration, root contact cannot be avoided completely. Thus, if necessary, insertion at HB15 in U6D could be executed at a gingival inclination of 70° using a thin miniscrew to protect the root, and additional distal inclination will be helpful, according to our results.

For each insertion point, we set paths at a distal inclination of 15° and 30° by rotating the initial coronal slices distally at 15° and 30° with the insertion point as the center and emanating paths in two new slices. Differing from the usual rectangular coordinate system, this method was built on a polar coordinate system and has the advantage of implementing measurements and applying its developed paths to clinical practice by rotating the handle distally and then elevating gingivally at the specific angle.

When distal inclination was added to the insertion path, bone depth increased and the bone deficiency ratio decreased, corroborating the findings of Du et al. [20]. Furthermore, the distal inclination added to the insertion path can partly improve bone availability and help prevent the distobuccal root of U6 in the U6D or U67 region, but the improvement is limited at the HB17 level. For the 0% bone deficiency ratio and high bone depth, combined with feasibility, the insertion path at a HB13 height in U7 at a gingival inclination of 70° and a distal inclination of 30° was preferred. Clinically, there is no need to correct the mesial inclined handle caused by the limited width of the mouth to strictly perpendicular to the buccal surface of the maxillary posterior region, but the inclination should be controlled.

## 5 Limitations

The lack of clinical soft tissue data in this retrospective tomography research limited the selection of miniscrew dimensions for the recommended paths in the IZC region. Another limitation was the use of a simple measurement line to detect bone depth, which could be substituted by a 3D miniscrew model to supplement our observations and provide more clinically relevant information in further studies. Furthermore, a retrospective review of different insertion paths in previous cases and clinical trials with larger sample sizes to clinically apply the results are needed to verify the conclusion.

## 6 Conclusions

Within the limits of this study, we can draw the following conclusions: (1) the U7M is an ideal extra-alveolar region in the IZC for inserting miniscrews, especially at the HB13 level; the paths at HB15 in the U7M are optional; (2) the U67 region has sufficient bone depth for miniscrew insertion at HB13 and HB15 height, but the buccal interradicular distance between U6 and U7 should be assessed preoperatively; (3) due to poor bone availability, the U6D region and height of HB17 are not recommended for miniscrew insertion; (4) the gingival and distal inclinations of the miniscrew could be properly increased to facilitate insertion, and a gingival inclination of 70° and a distal inclination of 30° could be beneficial, but the insertion should be performed only after careful imaging evaluation and 3D path planning.

## Ethics statement

This study was reviewed and approved by Ethics Committee of the Zhongshan Hospital of Fudan University (Xiamen), with the approval number: No. B2022-012.

Each participant provided written informed consent allowing their CBCT data to be used for research purposes and published anonymously.

## Funding statement

This research did not receive any specific grant from any public, commercial, or not-for-profit funding agencies for this research.

## Data availability statement

All data used in the generation of the results presented in this manuscript will be made available upon reasonable request from the corresponding author.

## CRediT authorship contribution statement

**Yingdan Pan:** Writing – original draft, Visualization, Methodology, Conceptualization. **Lijun Wei:** Writing – review & editing, Methodology, Investigation, Data curation. **Zhanglong Zheng:** Software, Formal analysis, Data curation. **Wei Bi:** Writing – review & editing, Supervision.

## Declaration of competing interest

The authors declare that they have no known competing financial interests or personal relationships that could have appeared to influence the work reported in this paper.

## Abbreviations

3D: three-dimensional
IZC: infrazygomatic crest
CBCT: cone beam computed tomography
U6: maxillary first molar
U7: maxillary second molar
U6D: distobuccal root of the maxillary first molar
U67: between the maxillary first molar and the maxillary second molar
U7M: mesiobuccal root of the maxillary second molar
FMA: Frankfurt mandibular plane angle
HB: horizontal base
HB13: 13 mm above the horizontal base plane
HB15: 15 mm above the horizontal base plane
HB17: 17 mm above the horizontal base plane
IP: insertion point
IP13: insertion point at 13 mm above the horizontal base plane
IP15: insertion point at 15 mm above the horizontal base plane
IP17: insertion point at 17 mm above the horizontal base plane
