# Supplementary material for: An evaluation of bone depth at different three-dimensional paths in infrazygomatic crest region for miniscrew insertion: A cone beam computed tomography study
Source: Heliyon. 2024 Feb 3;10(3):e25827. doi: 10.1016/j.heliyon.2024.e25827 (PMC10863323; doi:10.1016/j.heliyon.2024.e25827)
Supplement: Multimedia component 1 [file mmc1.docx]

Table S1. The median values of the bone depth (mm) at different insertion paths in male and female patients

| Sex | | | | | | | | Male | Female | P Value |
| --- | --- | --- | --- | --- | --- | --- | --- | --- | --- | --- |
| Number | | | | | | | | 76 | 84 |  |
| Insertion region | U6D | Insertion height | HB13 | Gingival inclination | 60° | Distal inclination | 0° | 3.93 | 3.97 | 0.82 |
|  |  |  |  |  |  |  | 15° | 4.98 | 4.84 | 0.88 |
|  |  |  |  |  |  |  | 30° | 6.43 | 6.71 | 0.72 |
|  |  |  |  |  | 70° | Distal inclination | 0° | 5.63 | 5.92 | 0.72 |
|  |  |  |  |  |  |  | 15° | 6.05 | 6.49 | 0.44 |
|  |  |  |  |  |  |  | 30° | 7.18 | 7.43 | 0.28 |
|  |  |  |  |  | 80° | Distal inclination | 0° | 6.63 | 7.34 | 0.22 |
|  |  |  |  |  |  |  | 15° | 7.15 | 7.88 | 0.08 |
|  |  |  |  |  |  |  | 30° | 8.34 | 8.95 | 0.06 |
|  |  |  | HB15 | Gingival inclination | 60° | Distal inclination | 0° | 4.08 | 4.12 | 0.79 |
|  |  |  |  |  |  |  | 15° | 4.13 | 4.25 | 0.76 |
|  |  |  |  |  |  |  | 30° | 4.97 | 5.63 | 0.99 |
|  |  |  |  |  | 70° | Distal inclination | 0° | 4.53 | 4.89 | 0.90 |
|  |  |  |  |  |  |  | 15° | 4.65 | 5.08 | 0.86 |
|  |  |  |  |  |  |  | 30° | 5.97 | 6.25 | 0.90 |
|  |  |  |  |  | 80° | Distal inclination | 0° | 4.93 | 5.59 | 0.40 |
|  |  |  |  |  |  |  | 15° | 5.10 | 5.98 | 0.35 |
|  |  |  |  |  |  |  | 30° | 6.47 | 7.14 | 0.25 |
|  |  |  | HB17 | Gingival inclination | 60° | Distal inclination | 0° | 3.39 | 3.54 | 0.76 |
|  |  |  |  |  |  |  | 15° | 3.41 | 3.37 | 0.85 |
|  |  |  |  |  |  |  | 30° | 3.58 | 4.11 | 0.56 |
|  |  |  |  |  | 70° | Distal inclination | 0° | 3.46 | 4.01 | 0.32 |
|  |  |  |  |  |  |  | 15° | 3.52 | 3.89 | 0.41 |
|  |  |  |  |  |  |  | 30° | 3.79 | 4.46 | 0.32 |
|  |  |  |  |  | 80° | Distal inclination | 0° | 3.82 | 4.36 | 0.23 |
|  |  |  |  |  |  |  | 15° | 3.91 | 4.37 | 0.27 |
|  |  |  |  |  |  |  | 30° | 4.32 | 4.83 | 0.21 |
|  | U67 | Insertion height | HB13 | Gingival inclination | 60° | Distal inclination | 0° | 6.58 | 6.11 | 0.21 |
|  |  |  |  |  |  |  | 15° | 7.10 | 7.00 | 0.95 |
|  |  |  |  |  |  |  | 30° | 7.28 | 7.13 | 0.23 |
|  |  |  |  |  | 70° | Distal inclination | 0° | 6.72 | 6.94 | 0.74 |
|  |  |  |  |  |  |  | 15° | 7.41 | 8.08 | 0.52 |
|  |  |  |  |  |  |  | 30° | 8.60 | 8.78 | 0.99 |
|  |  |  |  |  | 80° | Distal inclination | 0° | 7.12 | 7.70 | 0.17 |
|  |  |  |  |  |  |  | 15° | 7.96 | 8.96 | 0.25 |
|  |  |  |  |  |  |  | 30° | 9.43 | 10.75 | 0.95 |
|  |  |  | HB15 | Gingival inclination | 60° | Distal inclination | 0° | 4.84 | 5.08 | 0.82 |
|  |  |  |  |  |  |  | 15° | 5.31 | 5.63 | 0.99 |
|  |  |  |  |  |  |  | 30° | 6.20 | 6.52 | 0.95 |
|  |  |  |  |  | 70° | Distal inclination | 0° | 5.08 | 5.46 | 0.36 |
|  |  |  |  |  |  |  | 15° | 5.60 | 6.06 | 0.56 |
|  |  |  |  |  |  |  | 30° | 6.52 | 7.59 | 0.67 |
|  |  |  |  |  | 80° | Distal inclination | 0° | 5.69 | 5.92 | 0.21 |
|  |  |  |  |  |  |  | 15° | 6.21 | 6.60 | 0.29 |
|  |  |  |  |  |  |  | 30° | 7.07 | 8.81 | 0.23 |
|  |  |  | HB17 | Gingival inclination | 60° | Distal inclination | 0° | 3.67 | 4.02 | 0.57 |
|  |  |  |  |  |  |  | 15° | 3.75 | 3.90 | 0.66 |
|  |  |  |  |  |  |  | 30° | 4.22 | 4.50 | 0.79 |
|  |  |  |  |  | 70° | Distal inclination | 0° | 3.83 | 4.18 | 0.36 |
|  |  |  |  |  |  |  | 15° | 4.00 | 4.33 | 0.53 |
|  |  |  |  |  |  |  | 30° | 4.43 | 5.03 | 0.60 |
|  |  |  |  |  | 80° | Distal inclination | 0° | 4.16 | 4.55 | 0.22 |
|  |  |  |  |  |  |  | 15° | 4.20 | 4.66 | 0.34 |
|  |  |  |  |  |  |  | 30° | 5.01 | 5.79 | 0.40 |
|  | U7M | Insertion height | HB13 | Gingival inclination | 60° | Distal inclination | 0° | 6.42 | 6.54 | 0.69 |
|  |  |  |  |  |  |  | 15° | 7.47 | 7.78 | 0.73 |
|  |  |  |  |  |  |  | 30° | 7.56 | 7.73 | 0.83 |
|  |  |  |  |  | 70° | Distal inclination | 0° | 7.38 | 8.01 | 0.44 |
|  |  |  |  |  |  |  | 15° | 8.53 | 9.05 | 0.58 |
|  |  |  |  |  |  |  | 30° | 8.96 | 9.36 | 0.73 |
|  |  |  |  |  | 80° | Distal inclination | 0° | 7.74 | 8.56 | 0.21 |
|  |  |  |  |  |  |  | 15° | 9.31 | 10.12 | 0.24 |
|  |  |  |  |  |  |  | 30° | 10.22 | 11.39 | 0.45 |
|  |  |  | HB15 | Gingival inclination | 60° | Distal inclination | 0° | 5.63 | 5.63 | 0.54 |
|  |  |  |  |  |  |  | 15° | 6.24 | 6.38 | 0.88 |
|  |  |  |  |  |  |  | 30° | 7.23 | 7.15 | 0.98 |
|  |  |  |  |  | 70° | Distal inclination | 0° | 6.05 | 6.45 | 0.45 |
|  |  |  |  |  |  |  | 15° | 6.92 | 7.24 | 0.48 |
|  |  |  |  |  |  |  | 30° | 7.73 | 8.20 | 0.89 |
|  |  |  |  |  | 80° | Distal inclination | 0° | 6.36 | 7.02 | 0.25 |
|  |  |  |  |  |  |  | 15° | 7.53 | 8.05 | 0.20 |
|  |  |  |  |  |  |  | 30° | 8.40 | 9.73 | 0.45 |
|  |  |  | HB17 | Gingival inclination | 60° | Distal inclination | 0° | 4.29 | 4.76 | 0.87 |
|  |  |  |  |  |  |  | 15° | 4.55 | 4.87 | 0.43 |
|  |  |  |  |  |  |  | 30° | 5.18 | 5.87 | 0.32 |
|  |  |  |  |  | 70° | Distal inclination | 0° | 4.58 | 4.92 | 0.48 |
|  |  |  |  |  |  |  | 15° | 4.72 | 5.38 | 0.34 |
|  |  |  |  |  |  |  | 30° | 5.58 | 6.68 | 0.28 |
|  |  |  |  |  | 80° | Distal inclination | 0° | 4.90 | 5.29 | 0.29 |
|  |  |  |  |  |  |  | 15° | 5.18 | 5.93 | 0.20 |
|  |  |  |  |  |  |  | 30° | 6.28 | 7.90 | 0.17 |

U6D, distobuccal root of the maxillary first molar; U67, between the maxillary first molar and the maxillary second molar; U7M, mesiobuccal root of the maxillary second molar; HB, horizontal base; HB13, 13mm above the horizontal base plane; HB15, 15mm above the horizontal base plane; HB17, 17mm above the horizontal base plane
