# Supplementary material for: An evaluation of bone depth at different three-dimensional paths in infrazygomatic crest region for miniscrew insertion: A cone beam computed tomography study
Source: Heliyon. 2024 Feb 3;10(3):e25827. doi: 10.1016/j.heliyon.2024.e25827 (PMC10863323; doi:10.1016/j.heliyon.2024.e25827)
Supplement: Multimedia component 2 [file mmc2.docx]

Sup Table 2. The median values of the bone depth (mm) at different insertion paths on the left and right sides

| Insertion Side | | | | | | | | Left | Right | P value |
| --- | --- | --- | --- | --- | --- | --- | --- | --- | --- | --- |
| Insertion region | U6D | Insertion height | HB13 | Gingival inclination | 60° | Distal inclination | 0° | 4.06 | 3.91 | 0.67 |
|  |  |  |  |  |  |  | 15° | 5.20 | 4.71 | 0.48 |
|  |  |  |  |  |  |  | 30° | 6.89 | 6.14 | 0.32 |
|  |  |  |  |  | 70° | Distal inclination | 0° | 5.76 | 5.59 | 0.22 |
|  |  |  |  |  |  |  | 15° | 6.56 | 6.28 | 0.29 |
|  |  |  |  |  |  |  | 30° | 7.93 | 7.85 | 0.83 |
|  |  |  |  |  | 80° | Distal inclination | 0° | 7.18 | 6.64 | 0.08 |
|  |  |  |  |  |  |  | 15° | 7.76 | 7.33 | 0.33 |
|  |  |  |  |  |  |  | 30° | 9.09 | 9.20 | 0.73 |
|  |  |  | HB15 | Gingival inclination | 60° | Distal inclination | 0° | 4.15 | 4.04 | 0.40 |
|  |  |  |  |  |  |  | 15° | 4.20 | 4.18 | 0.18 |
|  |  |  |  |  |  |  | 30° | 5.64 | 5.31 | 0.29 |
|  |  |  |  |  | 70° | Distal inclination | 0° | 4.86 | 4.53 | 0.45 |
|  |  |  |  |  |  |  | 15° | 5.07 | 4.84 | 0.52 |
|  |  |  |  |  |  |  | 30° | 6.36 | 5.91 | 0.14 |
|  |  |  |  |  | 80° | Distal inclination | 0° | 5.52 | 5.06 | 0.18 |
|  |  |  |  |  |  |  | 15° | 5.98 | 5.53 | 0.06 |
|  |  |  |  |  |  |  | 30° | 7.04 | 6.69 | 0.11 |
|  |  |  | HB17 | Gingival inclination | 60° | Distal inclination | 0° | 3.57 | 3.40 | 0.14 |
|  |  |  |  |  |  |  | 15° | 3.44 | 3.26 | 0.46 |
|  |  |  |  |  |  |  | 30° | 4.08 | 3.59 | 0.19 |
|  |  |  |  |  | 70° | Distal inclination | 0° | 4.01 | 3.50 | 0.25 |
|  |  |  |  |  |  |  | 15° | 3.89 | 3.52 | 0.32 |
|  |  |  |  |  |  |  | 30° | 4.31 | 4.07 | 0.82 |
|  |  |  |  |  | 80° | Distal inclination | 0° | 4.35 | 3.89 | 0.10 |
|  |  |  |  |  |  |  | 15° | 4.28 | 4.04 | 0.07 |
|  |  |  |  |  |  |  | 30° | 4.64 | 4.68 | 0.58 |
|  | U67 | Insertion height | HB13 | Gingival inclination | 60° | Distal inclination | 0° | 6.48 | 6.33 | 0.39 |
|  |  |  |  |  |  |  | 15° | 7.22 | 6.80 | 0.07 |
|  |  |  |  |  |  |  | 30° | 7.50 | 7.00 | 0.92 |
|  |  |  |  |  | 70° | Distal inclination | 0° | 6.86 | 6.59 | 0.11 |
|  |  |  |  |  |  |  | 15° | 7.71 | 7.81 | 0.20 |
|  |  |  |  |  |  |  | 30° | 8.80 | 8.44 | 0.09 |
|  |  |  |  |  | 80° | Distal inclination | 0° | 7.25 | 7.42 | 0.08 |
|  |  |  |  |  |  |  | 15° | 8.32 | 8.44 | 0.21 |
|  |  |  |  |  |  |  | 30° | 10.27 | 10.05 | 0.35 |
|  |  |  | HB15 | Gingival inclination | 60° | Distal inclination | 0° | 5.02 | 4.71 | 0.28 |
|  |  |  |  |  |  |  | 15° | 5.54 | 5.32 | 0.26 |
|  |  |  |  |  |  |  | 30° | 6.43 | 6.09 | 0.08 |
|  |  |  |  |  | 70° | Distal inclination | 0° | 5.45 | 4.99 | 0.08 |
|  |  |  |  |  |  |  | 15° | 5.82 | 5.80 | 0.72 |
|  |  |  |  |  |  |  | 30° | 7.11 | 6.61 | 0.33 |
|  |  |  |  |  | 80° | Distal inclination | 0° | 5.82 | 5.69 | 0.41 |
|  |  |  |  |  |  |  | 15° | 6.39 | 6.24 | 0.67 |
|  |  |  |  |  |  |  | 30° | 7.93 | 8.11 | 0.34 |
|  |  |  | HB17 | Gingival inclination | 60° | Distal inclination | 0° | 3.90 | 3.90 | 0.72 |
|  |  |  |  |  |  |  | 15° | 3.86 | 3.80 | 0.44 |
|  |  |  |  |  |  |  | 30° | 4.31 | 4.19 | 0.35 |
|  |  |  |  |  | 70° | Distal inclination | 0° | 4.00 | 4.09 | 0.10 |
|  |  |  |  |  |  |  | 15° | 4.17 | 4.03 | 0.52 |
|  |  |  |  |  |  |  | 30° | 4.87 | 4.65 | 0.33 |
|  |  |  |  |  | 80° | Distal inclination | 0° | 4.29 | 4.36 | 0.93 |
|  |  |  |  |  |  |  | 15° | 4.54 | 4.46 | 0.44 |
|  |  |  |  |  |  |  | 30° | 5.61 | 5.24 | 0.18 |
|  | U7M | Insertion height | HB13 | Gingival inclination | 60° | Distal inclination | 0° | 6.48 | 6.55 | 0.72 |
|  |  |  |  |  |  |  | 15° | 7.67 | 7.74 | 0.77 |
|  |  |  |  |  |  |  | 30° | 7.60 | 7.63 | 0.68 |
|  |  |  |  |  | 70° | Distal inclination | 0° | 7.76 | 7.57 | 0.29 |
|  |  |  |  |  |  |  | 15° | 8.86 | 9.04 | 0.53 |
|  |  |  |  |  |  |  | 30° | 8.83 | 9.58 | 0.18 |
|  |  |  |  |  | 80° | Distal inclination | 0° | 8.12 | 8.21 | 0.08 |
|  |  |  |  |  |  |  | 15° | 9.65 | 9.81 | 0.14 |
|  |  |  |  |  |  |  | 30° | 10.36 | 10.98 | 0.52 |
|  |  |  | HB15 | Gingival inclination | 60° | Distal inclination | 0° | 5.70 | 5.57 | 0.37 |
|  |  |  |  |  |  |  | 15° | 6.19 | 6.39 | 0.68 |
|  |  |  |  |  |  |  | 30° | 7.28 | 7.14 | 0.11 |
|  |  |  |  |  | 70° | Distal inclination | 0° | 6.32 | 6.15 | 0.20 |
|  |  |  |  |  |  |  | 15° | 7.09 | 7.06 | 0.96 |
|  |  |  |  |  |  |  | 30° | 7.90 | 8.16 | 0.32 |
|  |  |  |  |  | 80° | Distal inclination | 0° | 6.58 | 6.81 | 0.57 |
|  |  |  |  |  |  |  | 15° | 7.69 | 7.67 | 0.10 |
|  |  |  |  |  |  |  | 30° | 9.04 | 9.48 | 0.42 |
|  |  |  | HB17 | Gingival inclination | 60° | Distal inclination | 0° | 4.64 | 4.50 | 0.53 |
|  |  |  |  |  |  |  | 15° | 4.77 | 4.76 | 0.72 |
|  |  |  |  |  |  |  | 30° | 5.42 | 5.63 | 0.26 |
|  |  |  |  |  | 70° | Distal inclination | 0° | 4.70 | 4.67 | 0.51 |
|  |  |  |  |  |  |  | 15° | 4.97 | 5.14 | 0.33 |
|  |  |  |  |  |  |  | 30° | 5.95 | 6.29 | 0.58 |
|  |  |  |  |  | 80° | Distal inclination | 0° | 5.08 | 5.25 | 0.72 |
|  |  |  |  |  |  |  | 15° | 5.48 | 5.61 | 0.65 |
|  |  |  |  |  |  |  | 30° | 6.74 | 7.11 | 0.07 |

U6D, distobuccal root of the maxillary first molar; U67, between the maxillary first molar and the maxillary second molar; U7M, mesiobuccal root of the maxillary second molar; HB, horizontal base; HB13, 13mm above the horizontal base plane; HB15, 15mm above the horizontal base plane; HB17, 17mm above the horizontal base plane
